# Lay and general practitioner attitudes towards endometrial cancer prevention: a cross-sectional study

**DOI:** 10.1093/fampra/cmad076

**Published:** 2023-07-28

**Authors:** Sarah J Kitson, Urwaa Khan, Emma J Crosbie

**Affiliations:** Division of Cancer Sciences, University of Manchester, Manchester, UK; Division of Cancer Sciences, University of Manchester, Manchester, UK; Division of Cancer Sciences, University of Manchester, Manchester, UK; Department of Obstetrics and Gynaecology, St Mary’s Hospital, Manchester University NHS Foundation Trust, Manchester Academic Health Science Centre, Manchester, UK

**Keywords:** attitudes, endometrial cancer prevention, general practice, women

## Abstract

**Background:**

Effective and targeted endometrial cancer prevention strategies could reduce diagnoses by 60%. Whether this approach is acceptable to individuals and general practitioners (GPs) is currently unknown. This study sought to determine attitudes towards the provision of personalised endometrial cancer risk assessments and the acceptability of potential prevention strategies.

**Methods:**

Specific online questionnaires were developed for individuals aged 45–60 years with a uterus and UK-practising GPs, with social media, charity websites, and email used to advertise the study. Individuals completed the questionnaires between February and April 2022.

**Results:**

Of 660 lay questionnaire respondents, 90.3% (*n* = 596) thought that undergoing an endometrial cancer risk assessment was a good or very good idea and 95.6% (*n* = 631) would be willing to undergo such an assessment. The commonest reasons for wanting to participate were “to try and reduce my risk” (*n* = 442, 67.0%), “to be informed” (*n* = 354, 53.6%), and “it could save my life’ (*n* = 315, 47.7%). Over 80% of respondents would make lifestyle changes to reduce their endometrial cancer risk (*n* = 550), with half accepting a pill, Mirena, or hysterectomy for primary prevention. GPs were similarly engaged, with 93.0% (*n* = 106) willing to offer an endometrial cancer risk assessment if a tool were available, potentially during a Well Woman screen.

**Conclusion:**

Personalised endometrial cancer risk assessments are acceptable to potentially eligible individuals and GPs and could be accommodated within routine practice. Clinical trials to determine the effectiveness of lifestyle modification and Mirena for endometrial protection are urgently required and should be targeted at those at greatest disease risk.

**Key messages**

- Women overwhelmingly want to know their personalised endometrial cancer risk.
- Over three quarters are willing to make lifestyle changes to reduce their risk.
- Half would accept a Mirena coil, tablet, or have a hysterectomy if needed.
- General Practitioners are keen to offer targeted endometrial cancer prevention.
- Tools to do so need to be developed and made available.

## Background

Endometrial cancer poses a significant health burden, with over 9,700 diagnoses annually in the United Kingdom, making it the fourth most common cancer in women.^1^ Diagnoses have risen by 59% over the last 3 decades as a result of changing demographics, namely an ageing society, rising rates of obesity, and fewer hysterectomies being performed for benign indications.^2^ This trend is unfortunately set to continue. By 2035, it is anticipated that 5% of all female malignancies in the United Kingdom will be due to endometrial cancer.^3^

While generally associated with an excellent prognosis due to early presentation with postmenopausal bleeding, 15% of women will have extrauterine disease at the time of diagnosis, which impacts significantly on survival.^4^ Endometrial cancer treatment can also impose a physical and psychological encumbrance on women, particularly those with obesity and associated co-morbidities which impact upon fitness for surgery and results in permanent loss of fertility in premenopausal women. There is also an associated financial cost, with data from a prospective nested cohort study within the UK Collaborative Trial of Ovarian Cancer Screening suggesting a cost of £10,000 for treating stage I endometrial cancer, according to 2013 prices, with mean costs of £27,270 over 5 years for women with stage IV disease.^5^

Given the strong association between endometrial cancer and modifiable risk factors, including obesity and physical inactivity, it should be an ideal target for primary prevention. Indeed, adherence to the American Cancer Society Guidelines of maintaining a healthy body weight, engaging in regular moderate to vigorous physical activity, making healthy dietary choices, and limiting alcohol intake could reduce the incidence of endometrial cancer by up to 60% (hazard ratio [HR] 0.40, 95% confidence interval [CI] 0.34–0.46).^6,7^ Despite a clear need for effective primary endometrial cancer prevention strategies, there has been relatively little work undertaken in this field to date.^8^ Exposure to the levonorgestrel-releasing intrauterine system is associated with a reduction in endometrial cancer incidence and is likely to be cost-effective in women with obesity, but only 1 prospective cohort study has been conducted to date to evaluate its efficacy in preventing endometrial cancer in asymptomatic women.^9–12^ This study enrolled 25 women with a body mass index (BMI) ≥ 40 kg/m^2^ and was able to show that the Mirena coil was both effective in reducing endometrial proliferation, as measured by Ki-67 immunohistochemical expression, as well as being acceptable to women, many of whom kept the coil beyond the end of the trial. Unfortunately, the POET trial, in which women with Lynch syndrome were to be randomised to a levonorgestrel-releasing intrauterine system or observation alone for 5 years, closed early due to poor recruitment.^13^ Data on the possible benefit of regular aspirin use have been conflicting, with the only randomised controlled trial insufficiently powered to detect a difference in the incidence of endometrial cancer between groups.^14,15^ A number of retrospective cohort studies of women who have undergone bariatric surgery have shown a beneficial effect of surgically induced weight loss on endometrial cancer risk, but the effect of more moderate weight loss through dietary modification and physical activity has yet to be established.^16^

For any primary prevention strategy to be effective it needs to be targeted at individuals at greatest risk of the disease. The development of an individualised endometrial cancer risk assessment tool and testing of interventions in those deemed at high risk of the disease certainly appeals to endometrial cancer survivors and those working in the field.^17^ Whether it is also acceptable to women without a history of endometrial cancer and general practitioners (GPs) who would be required to undertake the risk assessment and administer any interventions has yet to be determined. This study sought to determine the attitudes of women and GPs towards a risk-stratified approach to endometrial cancer prevention and, in so doing, provide critical data to inform future work aimed at developing an endometrial cancer risk prediction model and its use in clinical trials.

## Methods

A cross-sectional questionnaire study of lay individuals and GPs was conducted between 3 February 2022 and 24 April 2022 using the online platform Qualtrics. Two questionnaires were developed, one aimed at lay individuals and the other at GPs (Supplementary Appendix 1). Lay individuals were invited to participate if they were aged 45–60 years old, had a uterus, and had not previously been treated for endometrial cancer. GPs were required to be currently practicing in the United Kingdom. Questionnaires were developed through discussion among experts in endometrial cancer prevention and the patient voice panel of the Peaches womb cancer charity.

The study was publicised through the website, social media feeds, and emailing lists of the Wellbeing of Women and Peaches Womb Cancer Trust charities. GPs were contacted via the Primary Care Women’s Health forum and the Royal College of General Practitioners. Personal twitter accounts were also used to advertise the study to lay and professional followers.

Results from the Qualtrics website were downloaded *verbatim* and were uploaded in Stata (version 16) for analysis. Participant eligibility was checked against responses to questions of age (lay questionnaire) and geographical location (GP questionnaire) and ineligible participants removed.

## Analysis

A formal sample size calculation was not performed due to the nature of the study. Instead, participants were recruited until data saturation was reached, at which point no new information was gained. This was determined through interim analysis of both the closed text and free text responses after the study had been open for 1 and 2 months. Further advertisement of the study after 2 months elicited no further responses. The study team endeavoured to recruit individuals from across the United Kingdom to ensure generalisability of the results.

### Quantitative analysis

Responses to closed questions were analysed using a quantitative approach. Continuous data were summarised using median and interquartile ranges. Categorical data were summarised using percentages. No imputation of missing data were performed, instead these were reported as a separate category. Attitudes towards personalised endometrial cancer risk assessments and proposed interventions were analysed by ordinal logistic regression with the answer to the question as the dependent variable and the baseline characteristic as factor variables. A *P* value ≤ 0.05 was considered statistically significant.

### Qualitative analysis

Free-text boxes were included in the questionnaire to identify additional reasons why a particular response had been given aside from those already suggested by the authors and Peaches patient voice panel. Free-text comments were analysed using thematic analysis and an inductive approach, with coding and theme development driven by the responses given. Code lists were generated independently by Sarah Kitson (SK) and Urwaa Khan (UK) for each question with a free-text box. Lists were compared and refined until a consensus was reached. SK and UK worked together to apply the code list to the free-text responses.

## Results

There were 1,076 responses to the lay questionnaire, of which 416 were excluded due to failure to meet the inclusion criteria (*n* = 28) or to complete the acceptability questions (*n* = 388). This left 660 responses for the final analysis. There were 190 responses to the GP questionnaire, of which 75 were excluded due to failure to complete the acceptability questions. Three responses were excluded as they were from nonUK-practicing GPs, leaving 115 responses for analysis.

The baseline characteristics of study participants are shown in Table 1. Most respondents to the lay questionnaire were from the United Kingdom (*n* = 642, 97.3%) and were of White ethnicity (*n* = 622, 94.2%). Ninety percent (*n* = 593) were in paid employment and 77.5% (*n* = 506) had a college or university degree. One third (*n* = 216, 32.7%) of lay respondents had obesity (BMI ≥ 30 kg/m^2^) and 30.6% (*n* = 202) had been previously investigated for abnormal or postmenopausal bleeding. Participating GPs were also predominately female (*n* = 108, 93.9%) and of White ethnicity (*n* = 106, 92.2%). There was, however, good geographical coverage across the United Kingdom, with GPs working in a broad range of practice sizes.

**Table 1.** Baseline characteristics of study participants. a) Lay questionnaire b) GP questionnaire (2022).

Baseline characteristic	
a) Lay questionnaire (2022)	
Age yrs, median (IQR)	52 (48–56)
Age group, *n* (%)	
45-49	216 (32.7)
50–54	212 (32.1)
55–60	232 (35.2)
Ethnicity, *n* (%)	
White	622 (94.2)
Asian/Asian British	14 (2.1)
Black/African/Caribbean/Black British	10 (1.5)
Multiple ethnic groups	9 (1.4)
Other	5 (0.8)
Country of residence, *n* (%)	
UK	642 (97.3)
Region of UK	
London	71 (11.1)
North East	25 (3.9)
North West	90 (14.1)
Yorkshire	55 (8.6)
East Midlands	35 (5.5)
West Midlands	44 (6.9)
South East	107 (16.7)
East of England	49 (7.7)
South West	59 (9.2)
Wales	60 (9.4)
Scotland	38 (5.9)
Northern Ireland	7 (1.1)
Australia	4 (0.6)
France	1 (0.2)
Germany	1 (0.2)
Ireland	9 (1.4)
Mexico	2 (0.3)
Nigeria	1 (0.2)
Qualifications, *n* (%)	
GCSEs/NVQs or equivalent	65 (10.0)
A levels/AS levels or equivalent	72 (11.0)
College or university degree	506 (77.5)
None of the above	10 (1.5)
Employment, *n* (%)	
In paid employment/self-employed	593 (90.0)
Retired	19 (2.9)
Looking after home and/or family	17 (2.6)
Unable to work because of sickness or disability	11 (1.7)
Unemployed	3 (0.5)
Doing unpaid or voluntary work	10 (1.5)
Full- or part-time student	4 (0.6)
None of the above	2 (0.3)
Self-reported BMI kg/m^2^, median (IQR)	27.0 (23.3–31.6)
Menopausal status, *n* (%)	
Premenopausal	283 (42.9)
Postmenopausal	377 (57.1)
Parity, *n* (%)	
Nulliparous	140 (21.2)
1	102 (15.5)
2	275 (41.7)
3+	143 (21.7)
Current HRT use, *n* (%)	
Yes	257 (38.9)
No	403 (61.1)
Family history endometrial cancer, *n* (%)	
Yes	61 (9.2)
No	599 (90.8)
Family history bowel cancer, *n* (%)	
Yes	68 (10.3)
No	592 (89.7)
Previous investigations for abnormal vaginal bleeding/postmenopausal bleeding, *n* (%)	
Yes	202 (30.6)
No	458 (69.4)
b) GP questionnaire (2022)	
Gender, *n* (%)	
Female	108 (93.9)
Male	6 (5.2)
Non-binary	1 (0.9)
Age yrs, median (IQR)	45 (38–52)
Age group, *n* (%)	
30–39	30 (26.1)
40–49	48 (41.7)
50–59	29 (25.2)
60+	8 (7.0)
Ethnicity, *n* (%)	
White	106 (92.2)
Asian/Asian British	4 (3.5)
Black/African/Caribbean/Black British	2 (1.7)
Multiple ethnic groups	3 (2.6)
Region of UK, *n* (%)	
London	10 (8.8)
North East	3 (2.6)
North West	18 (15.8)
Yorkshire	9 (7.9)
East Midlands	9 (7.9)
West Midlands	9 (7.9)
South East	14 (12.3)
East of England	8 (7.0)
South West	13 (11.4)
Wales	9 (7.9)
Scotland	7 (6.1)
Northern Ireland	5 (4.3)
Practice size, *n* (%)	
1,000–4,999	7 (6.1)
5,000–9,999	45 (39.1)
10,000+	61 (53.0)
Locum	2 (1.7)
Number of GPs in the practice, *n* (%)	
1–5	25 (21.7)
6–10	65 (56.5)
11–15	17 (14.8)
16+	6 (5.2)
Locum	2 (1.7)
Family history endometrial cancer	
Yes, *n* (%)	7 (6.1)
No, *n* (%)	108 (93.9)

HRT, hormone replacement therapy.

**Fig. 1.**
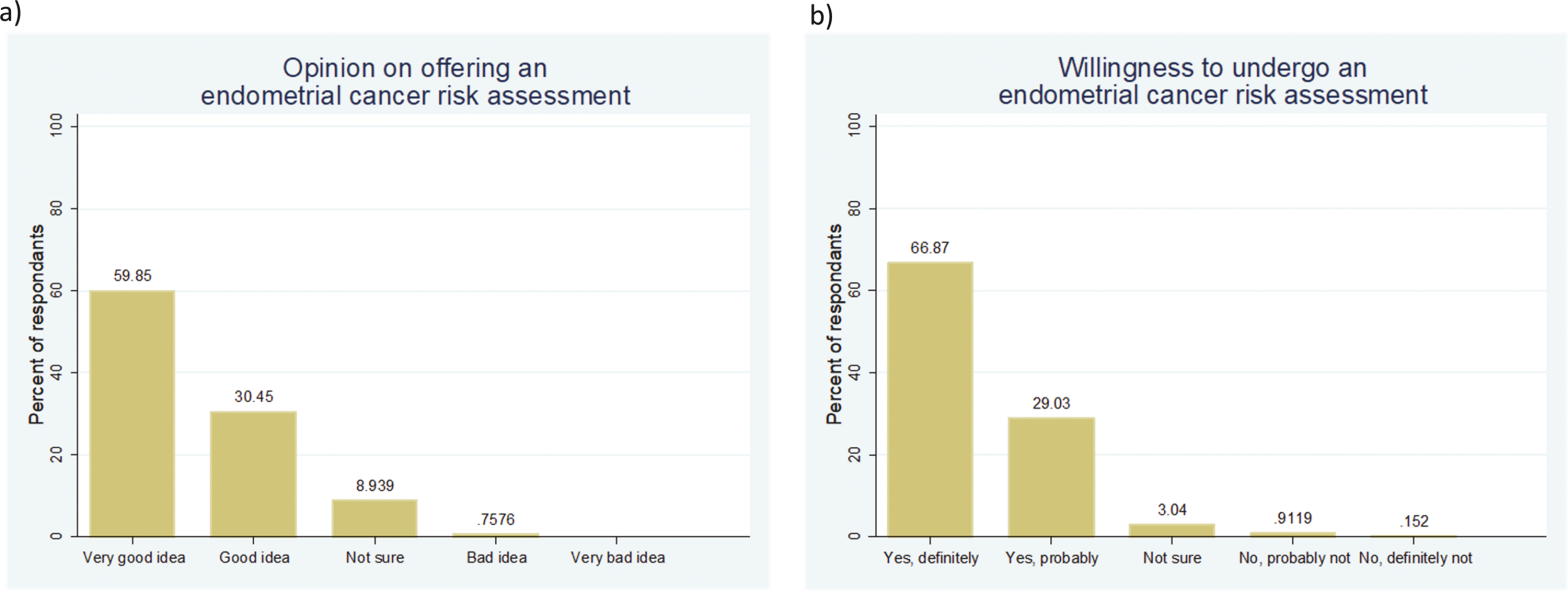
Attitudes of 600 lay respondents aged 45–60 years with an intact uterus to undergoing an individualised endometrial cancer risk assessment (2022). (a) Opinion on using factors like age, weight, lifestyle factors, medical history, reproductive history and family history to identify women at high risk of womb cancer, (b) personal willingness to have risk of womb cancer estimated using this information.

In total, 596 respondents to the lay questionnaire (90.3%) found the idea of undergoing an individualised endometrial cancer risk assessment to be a good or very good idea (Fig. 1a). Attitudes were unaffected by age, BMI, ethnicity, educational background, a family history of endometrial cancer or whether a respondent had previously been investigated for abnormal/postmenopausal bleeding (all *P* > 0.05). Six hundred and thirty-one individuals (95.6%) indicated that they would be willing to undergo such an assessment if a valid tool were available (Fig. 1b).

When asked why they had responded negatively towards undergoing an endometrial cancer risk assessment, reasons given included “there are no proven ways to reduce my risk” (*n* = 6, 0.9%), “it would cause too much worry” (*n* = 4, 0.6%), “it is not 100% accurate” (*n* = 4, 0.6%), “I am low risk/no family history” (*n* = 3, 0.5%), “it will affect health insurance” (*n* = 2, 0.3%), “would prefer a blood test to advice on diet and weight management” (*n* = 1, 0.2%), “I would rather not know” (*n* = 1, 0.2%), and “currently awaiting a hysterectomy” (*n* = 1, 0.2%). The most common reasons for expressing a desire to undergo an endometrial cancer risk assessment included “to allow me to try and reduce my risk/prevent endometrial cancer” (*n* = 442, 67.0%), “to be informed/so that I know” (*n* = 354, 53.6%), “it could save my life” (*n* = 315, 47.7%), and “to be reassured (that I am low risk)” (*n* = 233, 35.3%). Other stated reasons included “for research” (*n* = 222, 33.6%), “to save me from having to undergo treatment” (*n* = 212, 32.1%), “feel that doctors may look at any issue more seriously if this is a factor“ (*n* = 1, 0.2%), “just like other screening for prevention” (*n* = 1, 0.2%), “to be alert to potential indicators” (*n* = 1, 0.2%), and “to encourage others through my actions” (*n* = 1, 0.2%).

**Fig. 2.**
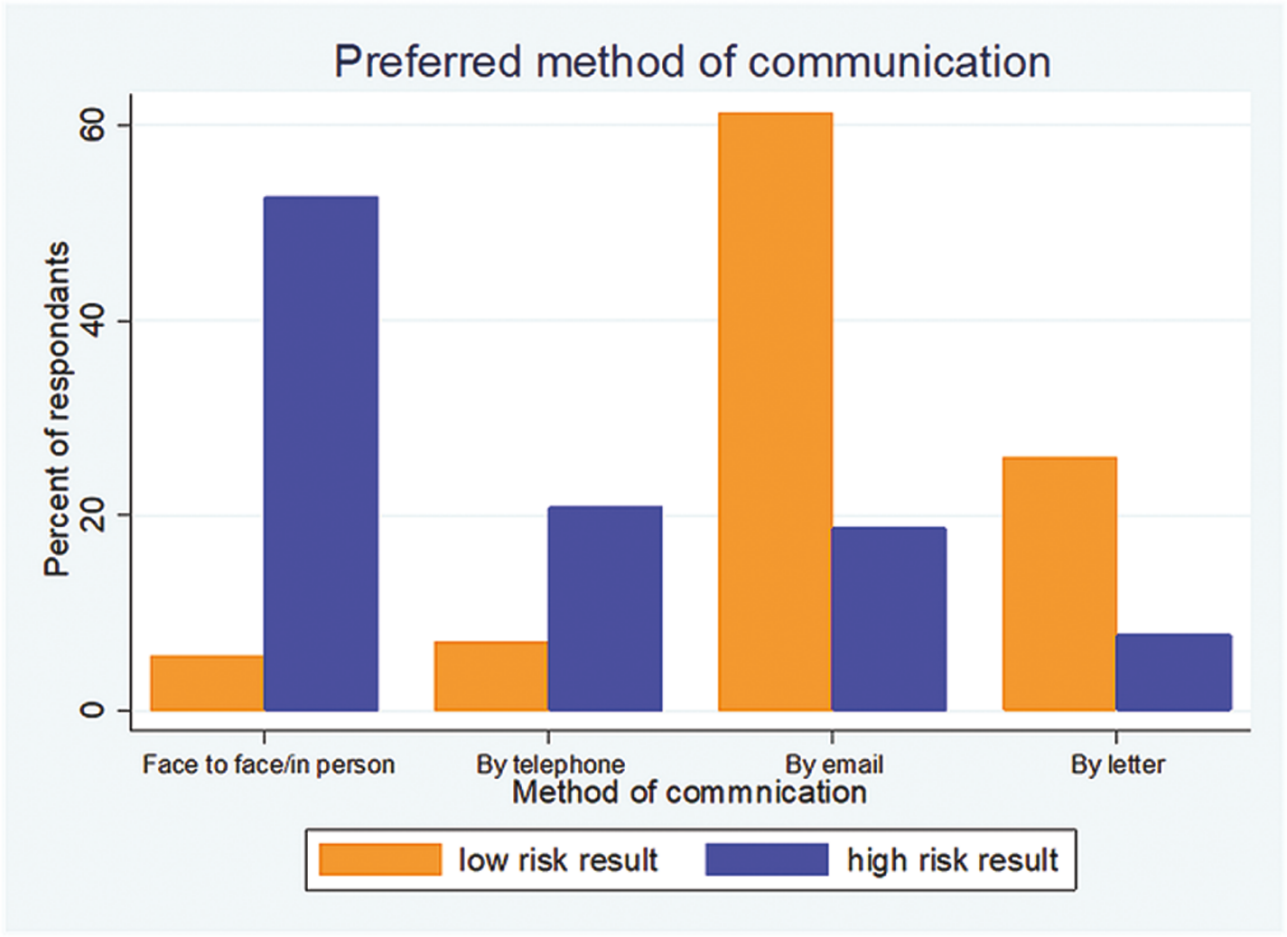
Preferred method of communication to lay respondent according to risk result (2022).

Respondents to the lay questionnaire had clear preferences as to how the findings of the risk assessment should be communicated (Fig. 2). If the result was low risk, 61.3% (*n* = 380) felt that this could be communicated by email and 26.0% (*n* = 161) preferred to be informed of the result by letter. In contrast, if the result was high risk, 52.6% (*n* = 331) of respondents preferred to receive this result in person.

Over 90% of respondents to the lay questionnaire would be willing to adopt one of the proposed prevention strategies if assessed to be at high risk of endometrial cancer. Lifestyle changes, including losing weight and increasing physical activity, was the most acceptable option, appealing to 83.3% (*n* = 550) of respondents. Taking a pill every day (*n* = 392, 59.4%), having a Mirena coil inserted (*n* = 317, 48.0%), or undergoing a hysterectomy (*n* = 337, 51.1%) were all of similar appeal. The commonest reasons for declining interventions were if they were deemed to be “invasive” (*n* = 91, 13.8%) or respondents were “worried about side effects” (*n* = 76, 11.5%). Other reasons included “difficulty in sustaining the intervention in the long term” (*n* = 14, 2.1%), “would prefer to discuss with a doctor first” (*n* = 11, 1.7%), “already having a Mirena in situ/exercising/being a healthy weight” (*n* = 5, 0.8%), “not wanting to take tablets” (*n* = 5, 0.8%), “feeling that a hysterectomy was a step too far” (*n* = 2, 0.3%), “contraindications to some options” (*n* = 1, 0.2%), “definitely would not want a coil” (*n* = 1, 0.2%), and “decision would be dependent upon factors in the risk assessment” (*n* = 1, 0.2%).

**Fig. 3.**
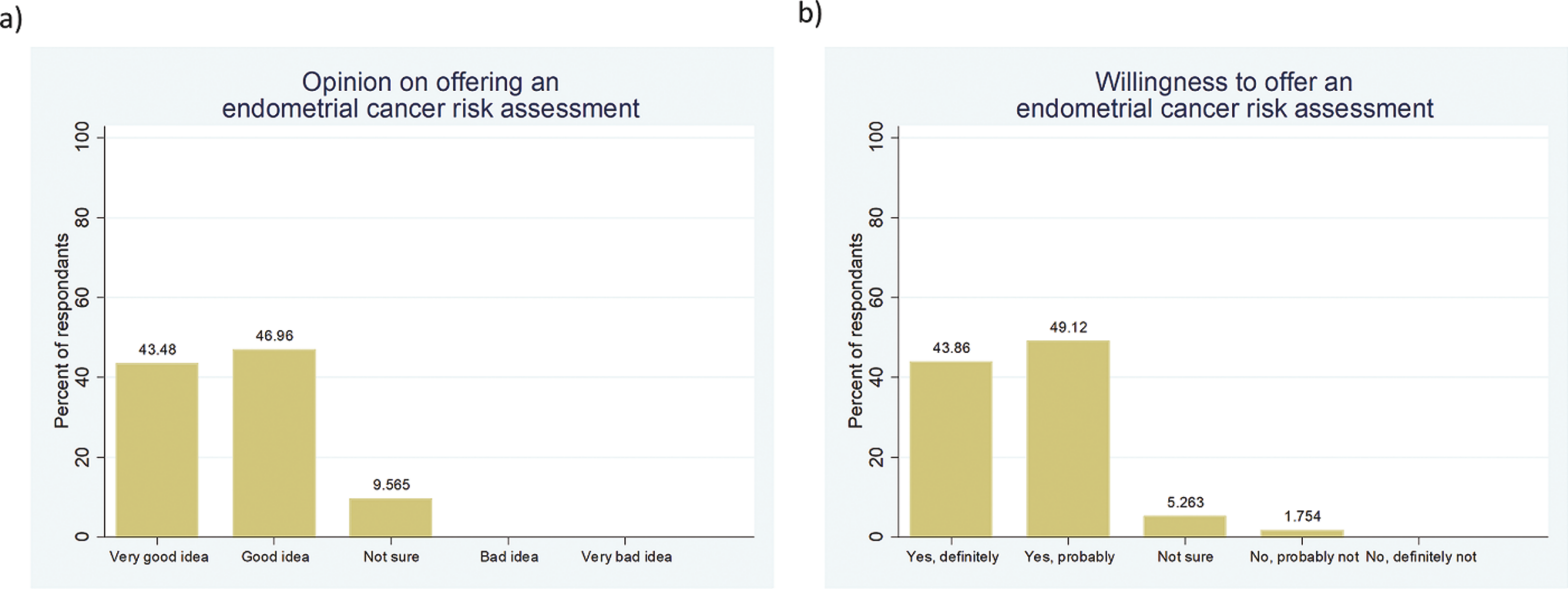
Attitudes of General Practitioners in the UK to offering an individualised endometrial cancer risk assessment (2022). (a) Opinion on using factors like age, weight, lifestyle factors, medical history, reproductive history and family history to identify women at high risk of endometrial cancer, (b) willingness to offer an endometrial cancer risk assessment to women aged 45–60 years if a validated risk assessment tool were available.

GPs were also in favour of using an endometrial cancer risk assessment to identify women at high risk of the disease. One hundred and four (90.3%) respondents thought the idea of using an endometrial cancer risk assessment tool was a good or very good idea (Fig. 3a). One hundred and six GPs (93.0%) would be willing to offer an endometrial cancer risk assessment if a valid tool were available (Fig. 3b). Reasons for declining included “a lack of time” (*n* = 4, 3.5%), “absence of effective prevention strategies” (*n* = 2, 1.7%), “a fear of causing their patients too much worry” (*n* = 1, 0.9%), “the potential impact on health insurance” (*n* = 1, 0.9%), and “a need for any harms to be outweighed by the benefits of the risk assessment” (*n* = 1, 0.9%). The most common reason for offering the risk assessment was that it would “help with shared decision making about how to reduce endometrial cancer risk” (*n* = 93, 80.9%). Other reasons included to “inform them and their patients of their endometrial cancer risk” (*n* = 73, 63.5%), “to save lives” (*n* = 58, 50.4%), “it would be cost-effective in the long term” (*n* = 47, 40.9%), “to allow research into endometrial cancer prevention” (*n* = 43, 37.4%), “for reassurance” (*n* = 20, 17.4%), “to be used as a secondary triage of symptoms of abnormal vaginal bleeding” (*n* = 2, 1.7%), and that “it could be sent out as a questionnaire via accurx and, therefore, not take up much clinician time” (*n* = 1, 0.9%).

Responding GPs felt that the endometrial cancer risk assessment would be best accommodated as part of a Well Woman screen (*n* = 64, 55.7%), in preference to being undertaken during a GP consultation (*n* = 15, 13.0%). Alternatively, it could be conducted by specialist nurses (*n* = 2, 1.7%), a component of long-term reviews (*n* = 1, 0.9%), or when managing a patient with abnormal bleeding to decide upon need for secondary care referral (*n* = 1, 0.9%). The use of remote questionnaires to be sent out to patients appealed to 7.8% (*n* = 9) of GPs. Over two thirds of GPs (*n* = 81, 71.7%) would be willing to recruit patients into clinical trials of endometrial cancer prevention strategies.

## Discussion

Potentially eligible individuals and currently practising GPs are overwhelmingly in favour of a personalised endometrial cancer risk assessment being made available and would make use of it to inform and influence decision making. A risk assessment tool needs to fit easily into routine clinical practice and be suitable for completion remotely, with immediate feedback of low-risk results and the offer of a face-to-face discussion with a clinician for those assessed to be at high risk. Individuals would be largely willing to make lifestyle changes, including losing weight and increasing their physical activity, if assessed to be at high risk of endometrial cancer, as well as potentially accepting of tablets, a Mirena intrauterine system, or undergoing a hysterectomy. The effectiveness of these nonsurgical interventions for the primary prevention of endometrial cancer should, therefore, be determined.

The strength of this study lies in its large sample size, with 660 lay individuals and 115 GPs completing the questionnaires, increasing the reliability and validity of the study’s findings. Responders to the lay questionnaire closely mirrored the total UK female population, with broad geographical coverage and similar rates of obesity (32.7% vs. 35%).^18^ A greater proportion of responders were of White ethnicity than are represented in the general population (study 94.2% vs. population 86%), potentially reflecting the fact that the questionnaire was promoted and completed online only. Similarly, responders to the GP survey were also predominately female (study 93.9% vs. population 57.1%) and of White ethnicity (study 92.2% vs. population 49.8%). This is likely to reflect the fact that the study was focussed on women’s health issues, with which female GPs were more likely to identify.

The study was publicised widely through social media and email lists of women’s health charities and the Royal College of General Practitioners with minimal exclusion criteria to encourage as many individuals as possible to complete the relevant questionnaires. The digital format may have limited accessibility for some, particularly Black, Asian, and Minority Ethnic groups who traditionally have lower response rates to online questionnaires and uptake of heath prevention programmes, including cervical screening.^19^ Specific engagement with the Black, Asian, and Minority Ethnic community needs to be undertaken to ensure that their needs are met and that the benefits of any future endometrial cancer prevention programme are available to all. Poor accessibility is unlikely to explain the lower response rate by nonWhite GPs though. Black, Asian, and Minority Ethnic GPs in the United Kingdom are more likely to work as locums than UK qualified White doctors and may not have been aware of the study if they were not included on the Royal College of General Practitioners’ email distribution list to research active clinicians. Whether the views of Black, Asian, and Minority Ethnic GPs differ from those reported here is unknown. A large number of individuals who opened the survey failed to complete the questions on acceptability and were, therefore, excluded from the study. Whilst the questionnaire was deliberately kept as short as possible and responses could be saved and returned to, some may have been put off completing it due to concerns about the time required to do so. This is a limitation of online surveys where the respondent is not in direct communication with the research team, meaning that we are unable to explore the reasons for noncompletion further. These individuals also did not complete the demographic questions so we are unable to comment on any differences in basic characteristics between responders and nonresponders. This study is also deliberately UK-centric, as our research team are looking to undertake further work in the field of primary endometrial cancer prevention within the context of the UK healthcare system. Whether the findings of this study are shared globally is unknown.

This is the first study to specifically focus on the attitudes of individuals within the general population and GPs towards endometrial cancer prevention. This is an area that has traditionally been underresearched, as highlighted by the James Lind Alliance under the “ Prevention and Screening Domain” of their endometrial cancer Research Priority Setting partnership in 2016.^17^ A previous survey of 74 women with class II and III obesity attending gynaecology, obesity, and sleep apnoea clinics in Manchester found that an even greater proportion of this very high-risk group of women would be willing to lose weight (94%) or take a pill every day (74%) to reduce their endometrial cancer risk compared with the general population surveyed in this study.^20^ Interestingly, the proportion of individuals willing to have a coil inserted for primary endometrial cancer prevention was identical in the 2 cohorts. The PROgesterone Therapy for Endometrial Cancer prevention in obese women (PROTEC) trial was able to document the actual uptake of the Mirena intrauterine system for endometrial protection in women with class-III obesity, finding that 65% of women approached would accept the insertion of the coil with 10% withdrawal due to the discomfort of the endometrial sampling procedure.^12^ Interestingly, once inserted, 24 of the 25 women opted to keep the Mirena coil in for long-term endometrial protection.

This study provides important information on the attitudes of potentially eligible individuals towards a personalised endometrial cancer risk assessment and primary disease prevention and covers those likely to be at both low and high risk of the disease. These data are encouraging that women within the general population are potentially more amenable to participating in risk-stratified endometrial cancer prevention trials than women with Lynch syndrome. Researchers involved in the POET trial have suggested that individuals at significantly elevated lifetime risk of endometrial cancer were polarised in their views as to what risk-reducing strategies they would be willing to accept, with women opting either for a hysterectomy once childbearing was complete or wanting a Mirena coil fitted, with few accepting of observation alone. Any future endometrial cancer prevention trial conducted at population level needs, therefore, to be carefully designed with a patient and public involvement group and is likely to necessitate that all women be exposed to an active intervention.

Based on the findings of this work, we propose to develop and validate an endometrial cancer risk prediction model for use in the UK population and to use this to determine eligibility for a risk-stratified endometrial cancer prevention trial of a levonorgestrel-releasing intrauterine system and lifestyle modification for endometrial protection. We propose to undertake a feasibility study initially to confirm that women are willing to be recruited into such a clinical trial. We hope with this to be able to demonstrate that such an approach is clinically and cost-effective, a necessary prerequisite to it being incorporated into routine general practice.

## Supplementary material

Supplementary material is available at *Family Practice* online.

cmad076_suppl_Supplementary_Data

cmad076_suppl_Supplementary_Material

## Data Availability

The data underlying this article will be shared on reasonable request to the corresponding author.
