# Supplementary material for: Lay and general practitioner attitudes towards endometrial cancer prevention: a cross-sectional study
Source: Fam Pract. 2023 Jul 28;41(6):949–55. doi: 10.1093/fampra/cmad076 (PMC11636559; doi:10.1093/fampra/cmad076)
Supplement: cmad076_suppl_Supplementary_Material [file cmad076_suppl_supplementary_material.pdf]

Supplementary method 1

Lay questionnaire

I consent to participate in this study (please tick) ☐

Demographic information

To which gender do you identify?

☐Male	☐Female	☐Non-binary	☐Prefer to self-describe
☐Prefer not to say			

How old are you? ☐

Which of the following best describes your ethnicity?

**White**	**Multiple ethnic groups**	**Asian/ Asian British**	**Black/African/Caribbean/ Black British**
☐British<br>☐Irish<br>☐Gypsy or Irish Traveller<br>☐White Other, please describe:	☐White and Black Caribbean<br>☐White and Black African<br>☐White and Asian<br>☐Multiple other, please describe:	☐Indian<br>☐Pakistani<br>☐Bangladeshi<br>☐Chinese<br>☐Asian other, please describe:	☐African<br>☐Caribbean<br>☐Black/African/Caribbean other, please describe:
**Other ethnic group**<br>☐Arab		☐Prefer not to say	

☐Other, please describe:	

Which country do you live in?

If you live in the UK, which region do you live in?

☐London	☐North East	☐North West	☐Yorkshire
☐ East Midlands	☐ West Midlands	☐ South East	☐ East of England
☐ South West	☐ Wales	☐ Scotland	☐ Northern Ireland

What is your highest qualification?

☐College or university degree	☐ A levels/AS levels or equivalent	☐GCSEs/NVQs or equivalent
☐ None of the above	☐Prefer not to say	

What is your current employment status? (you may chose more than one option)

	☐ Retired		

☐In paid employment/self-employed		☐Looking after home and/or family	☐Unable to work because of sickness or disability
☐ Unemployed	☐ Doing unpaid or voluntary work	☐Full or part-time student	☐None of the above
☐ Prefer not to say			

Personal and medical history

How tall are you? [Ft inches] or [cm]

How much do you weigh? [stones] or [kgs]

How old were you when you periods started? [ ]

Do you still have periods? ☐ Yes ☐ No

If not, how old were you when your periods stopped? [ ]

How many children do you have? [ ]

How old were you when your **LAST** child was born? [ ]

Do you currently use HRT? ☐ Yes ☐ No

Have you ever used the combined oral contraceptive pill for five or more years?

☐ Yes ☐ No

Do you currently take tamoxifen? ☐ Yes ☐ No

Do you have type 2 diabetes? ☐ Yes ☐ No

Do you or have you ever smoked? ☐ Yes ☐ No

Have any of your first degree relatives (mother, father, siblings, children) been diagnosed with bowel cancer?

Yes ☐ No ☐

Has anyone in your family been diagnosed with womb cancer?

Yes ☐ No ☐

Have you ever been investigated for abnormal vaginal/postmenopausal bleeding?

Yes ☐ No ☐

<u>Attitude towards a personalised assessment of endometrial (womb) cancer risk</u>

What is your opinion on using factors like age, weight, lifestyle factors, medical history, reproductive history and family history to identify women at high risk of womb cancer?

☐Very bad idea	☐Bad idea	☐Good idea	☐Very good idea
☐ Not sure		☐Prefer not to say	

Would you personally be willing to have your risk of womb cancer estimated using this information, if the NHS were to offer it?

☐No, definitely not	☐No, probably not	☐Yes, probably	☐Yes, definitely
☐ Not sure		☐Prefer not to say	

If you answered **NO** to undergoing a personalised risk assessment, why was this? (you may chose more than one option)

☐It would cause me too much worry	☐I would rather not know	☐I am low risk/no family history	☐It is not 100% accurate
☐ It will affect (health) insurance	☐ There are no proven ways to reduce my risk if it is high	☐Prefer not to say	Please add any other reason

If you answered **YES** to undergoing a personalised risk assessment, why was this? (you may chose more than one option)

☐To allow me to try and reduce my risk/prevent endometrial cancer	☐To be informed/so that I know	☐To be reassured (that I am low risk)	☐Could save my life

☐ To save me from having to undergo treatment for cancer in the future	☐ For research	☐Prefer not to say	Please add any other reason

How would you like to receive your personalised risk assessment result, if you were **LOW** risk?

☐Face to face/in person	☐By telephone	☐By email	☐By letter
☐ Not sure		☐Prefer not to say	

How would you like to receive your personalised risk assessment result, if you were **HIGH** risk?

☐Face to face/in person	☐By telephone	☐By email	☐By letter
☐ Not sure		☐Prefer not to say	

If you were assessed to be at **HIGH** risk of womb cancer, which of the following prevention strategies would you be willing to use to reduce your risk? (you may chose more than one)

☐Lifestyle changes (losing weight, increasing physical activity)	☐Take a pill every day	☐Have a Mirena (progesterone releasing) coil inserted

☐Undergo a hysterectomy (surgical removal of the womb)	☐ Not sure	☐Prefer not to say

If you have answered no to any of the above options, why was this? (you may chose more than one)

☐Too difficult to keep up long term	☐Invasive	☐Worried about side effects
☐ Prefer not to say	☐Other (please state)	

GP questionnaire

I consent to participate in this study (please tick) ☐

Demographic information

To which gender do you identify?

☐Male	☐Female	☐Non-binary	☐Prefer to self-describe
☐Prefer not to say			

How old are you?

Which of the following best describes your ethnicity?

**White**	**Multiple ethnic groups**	**Asian/ Asian British**	**Black/African/Caribbean/ Black British**
☐British<br>☐Irish<br>☐Gypsy or Irish Traveller<br>☐White Other, please describe:	☐White and Black Caribbean<br>☐White and Black African<br>☐White and Asian<br>☐Multiple other, please describe:	☐Indian<br>☐Pakistani<br>☐Bangladeshi<br>☐Chinese<br>☐Asian other, please describe:	☐African<br>☐Caribbean<br>☐Black/African/Caribbean other, please describe:
**Other ethnic group**<br>☐Arab<br><br>☐Other, please describe:		☐Prefer not to say	

Which country do you live in? ☐

If you live in the UK, which region do you live in?

☐London	☐North East	☐North West	☐Yorkshire
☐ East Midlands	☐ West Midlands	☐ South East	☐ East of England
☐ South West	☐ Wales	☐ Scotland	☐ Northern Ireland

Has anyone in your family been diagnosed with endometrial cancer?

☐ Yes ☐ No

Practice characteristics

What is your practice list size? ☐

How many GPs work in your practice? ☐

Attitude towards a personalised assessment of endometrial cancer risk

What is your opinion on using factors like age, weight, lifestyle factors, medical history, reproductive history and family history to identify women at high risk of endometrial cancer?

☐Very bad idea	☐Bad idea	☐Good idea	☐Very good idea
☐ Not sure		☐Prefer not to say	

Would you be willing to offer an endometrial cancer risk assessment to women aged 45-60 years in your practice if a validated risk assessment tool were available?

☐No, definitely not	☐No, probably not	☐Yes, probably	☐Yes, definitely
☐ Not sure		☐Prefer not to say	

If you answered **YES** to conducting endometrial cancer risk assessments, why was this? (you may chose more than one option)

☐It would inform me and my patients of their endometrial cancer risk	☐It would allow me and my patients to make decisions about how to reduce their endometrial cancer risk	☐It would reassure me and my patients	☐It could save lives

☐ It could be cost-effective in the long run	☐It would allow research into endometrial cancer prevention	☐Prefer not to say	Please add any other reason

If you answered **NO** to conducting endometrial cancer risk assessments, why was this? (you may chose more than one option)

☐It would cause my patients too much worry	☐I am already able to determine who is at high risk based on family history	☐My patients would rather not know	☐It is not 100% accurate	☐No time	☐ It would be too expensive
☐ It may affect (health) insurance	☐ There are no proven ways to reduce endometrial cancer risk if it is high	☐Not trained to do so	☐ Prefer not to say	Please add any other reason	

What would be the best way to conduct an endometrial cancer risk assessment?

☐As part of a GP clinic appointment	☐As part of a Well Woman check	☐ Not sure	☐Other

Would you be willing to recruit women into clinical trials of primary endometrial cancer prevention strategies?

☐ Yes ☐ No
